# A systematic review of the active saikosaponins and extracts isolated from Radix Bupleuri and their applications

**DOI:** 10.1080/13880209.2016.1262433

**Published:** 2016-12-12

**Authors:** Bochuan Yuan, Rui Yang, Yongsheng Ma, Shan Zhou, Xiaodong Zhang, Ying Liu

**Affiliations:** School of Chinese Pharmacy, Beijing University of Chinese Medicine, Beijing, China

**Keywords:** Radix Bupleuri, saikosaponins, anti-inflammatory, antitumor, neuroregulation

## Abstract

**Context:** Radix Bupleuri has been used in traditional Chinese medicine for over 2000 years with functions of relieving exterior syndrome, clearing heat, regulating liver-*qi*, and lifting yang-*qi*. More natural active compounds, especially saikosaponins, have been isolated from Radix Bupleuri, which possess various valuable pharmacological activities.

**Objective:** To summarize the current knowledge on pharmacological activities, mechanisms and applications of extracts and saikosaponins isolated from Radix Bupleuri, and obtain new insights for further research and development of Radix Bupleuri.

**Methods:** PubMed, Web of Science, Science Direct, Research Gate, Academic Journals and Google Scholar were used as information sources through the inclusion of the search terms ‘Radix Bupleuri’, ‘*Bupleurum*’, ‘saikosaponins’, ‘Radix Bupleuri preparation’, and their combinations, mainly from the year 2008 to 2016 without language restriction. Clinical preparations containing Radix Bupleuri were collected from official website of China Food and Drug Administration (CFDA).

**Results and conclusion:** 296 papers were searched and 128 papers were reviewed. A broad spectrum of *in vitro* and *in vivo* research has proved that Radix Bupleuri extracts, saikosaponin a, saikosaponin d, saikosaponin c, and saikosaponin b_2_, exhibit evident anti-inflammatory, antitumor, antiviral, anti-allergic, immunoregulation, and neuroregulation activities mainly through NF-*κ*B, MAPK or other pathways. 15 clinical preparations approved by CFDA remarkably broaden the application of Radix Bupleuri. The main side effect of Radix Bupleuri is liver damage when the dosage is excess, which indicates that the maximum tolerated dose is critical for clinical use of Radix Bupleuri extract and purified compounds.

## Introduction

**Figure 1.**
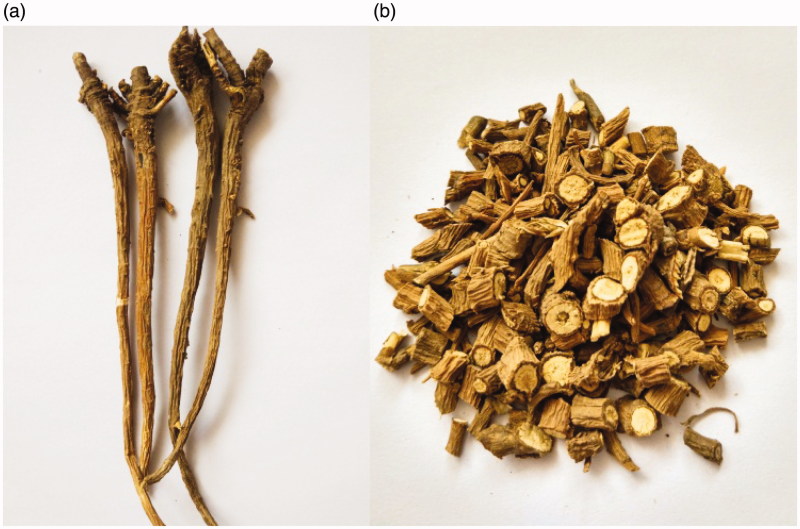
Radix Bupleuri (a) and its pieces (b).

With a 2000-year medicinal history, Radix Bupleuri (*Chai Hu* in Chinese) is believed to be one of the most important herbal medicines in China. The earliest record about Radix Bupleuri in China appeared in *Shen Nong Ben Cao Jing*, the first Chinese medical book, since then, Radix Bupleuri has been widely used in traditional Chinese medicine (TCM) for its effects of relieving exterior syndrome, clearing heat, regulating the liver-*qi*, and lifting yang-*qi* (Sen 1959). It has been used in many traditional Chinese prescriptions, such as *Xiao Chai Hu Tang* and *Chai Hu Shu Gan Yin* to treat cold and liver diseases (Chen et al. 2011). The roots are usually the medicinal parts of Radix Bupleuri, and which is often processed into pieces for easy use (Figure 1).

**Figure 2.**
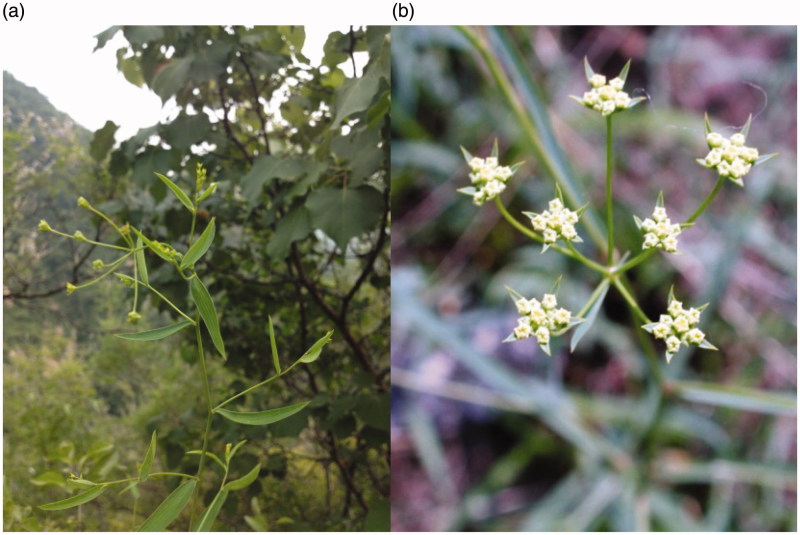
*Bupleurum chinense* DC. (a) Shows the compound umbels and simple, long, slender leaves, (b) shows the yellowish bisexual flowers of compound umbels.

*Bupleurum chinense* DC. (Apiaceae) and *Bupleurum scorzonerifolium* Willd. are defined as the original plants of Radix Bupleuri in *Chinese Pharmacopeia* (National Pharmacopoeia Committee 2010). In fact, many other *Bupleurum* species are also used as Radix Bupleuri in East Asia, such as *Bupleurum falcatum* L., which is officially listed in *Japanese Pharmacopeia* (Saiko in Japanese) (Japanese Pharmacopoeia Editorial Board 2011), and *Bupleurum yinchowense* Shan and Li, which is recorded in some provincial *Pharmacopeia* of China (The Inner Mongolia Autonomous Region Health Department 1988; Food and Drug Administration of Gansu Province 2008). These *Bupleurum* medicinal plants are widely distributed in the northern hemisphere (Judd 2008), and also commonly used in Eurasia and North Africa for their medicinal properties (Mabberley 2008). As shown in Figure 2, they are perennial herbs with compound umbels, yellowish or rarely purplish bisexual flowers, containing five stamens, cremocarps, and simple, long, slender leaves (Figure 2).

With the development of modern pharmacology, many valuable and important activities of Radix Bupleuri have been discovered, such as anti-inflammatory (Xie et al. 2012), antitumor (Liu & Li 2014), antidepressant (Jin et al. 2013), antiviral (Chiang et al. 2003), hepatoprotection (Wang et al. 2013a), immunoregulation (Ying et al. 2014), and neuromodulation activities (Zhou et al. 2014). All of these potent effects are due to its various secondary metabolites, especially saikosaponins, the content of which is up to 7% of the total dry weight of Radix Bupleuri roots (Ashour & Wink 2011). To date, over 100 glycosylated oleanane-type saponins have been isolated and identified from Radix Bupleuri (Pistelli et al. 1993; Ebata et al. 1996), and some of them have been demonstrated possessing bioactive properties both *in vitro* and *in vivo*. Therefore, reviewing and summarizing the pharmacological activities and mechanisms of saikosaponins from Radix Bupleuri is meaningful and important to obtain new insights for further research and development of Radix Bupleuri. In addition, since extracts are the main source of Chinese patent medicines containing Radix Bupleuri, their pharmacological properties and mechanisms are also summarized. Moreover, the applications and toxicity studies are discussed to provide a basis for further studies concerning the safety and efficacy of Radix Bupleuri.

In this paper, six main databases, PubMed, Web of Science, Science Direct, Research Gate, Academic Journals, and Google Scholar were used as information sources through the inclusion of the search terms ‘Radix Bupleuri’, ‘*Bupleurum*’, ‘saikosaponins’, ‘Radix Bupleuri preparation’, and their combinations, mainly from the year 2008 to 2016 without language restriction. As a result, we searched 296 papers and a total of 128 references were included in the present work.

## Purified saikosaponins from Radix Bupleuri

**Figure 3.**
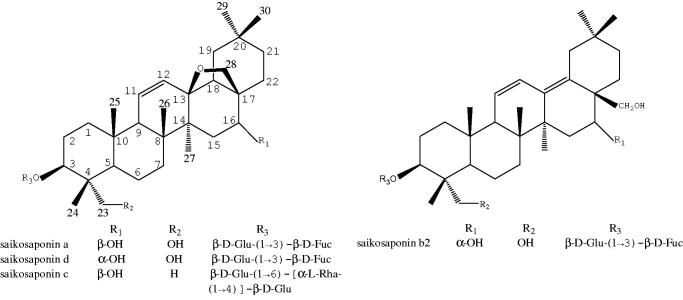
The structures of SSa, SSd, SSc and SSb_2_.

In recent years, over 100 different triterpenoid saponins have been isolated from Radix Bupleuri, among them saikosaponin a (SSa), saikosaponin d (SSd), saikosaponin c (SSc) and saikosaponin b_2_ (SSb_2_) (Figure 3) are believed to be responsible for the most pharmacological activites of Radix Bupleuri (Liu et al. 2002; Huang et al. 2013). Saikosaponins are oleanane type triterpenoid saponins and divided into seven types according to different aglycones. SSa, SSd and SSc are epoxy-ether saikosaponins (type I), while SSb_2_, with a different aglycone, is heterocyclic diene saikosaponin (type II) (Lin et al. 2013).

## SSa

SSa, one of the most important active saikosaponins in Radix Bupleuri (Liang et al. 2014), plays a significant role in anti-inflammatory (Wu et al. 2008, 2010; Han et al. 2011; Lu et al. 2012b; Chen et al. 2013b; Wang et al. 2013b; Zhu et al. 2013; Fu et al. 2015; Kim et al. 2015; Zhao et al. 2015a; Zhou et al. 2015), antitumor (Tsai et al. 2002; Wang et al. 2010a, 2010b), antiviral (Cheng et al. 2006; Chen et al. 2015), neuromodulation (Yu et al. 2012; Xie et al. 2013; Yoon et al. 2012, 2013; Zhou et al. 2014), and immunoregulation (Sun et al. 2009) activities. The various pharmacological activities, mechanisms, models and applications of SSa are given in Table 1.

**Table 1.** The various pharmacological activities, mechanisms, models, and applications of SSa.

Pharmacologicalactivities of SSa	Tissue	Models/cells	*In vivo*/*vitro*	Mechanisms	Applications	References
Anti-inflammatory activity	Adipocytes	3T3-L1	*In vitro*	SSa inhibits the expression of inflammatory associated genes and is a potent inhibitor of NF-*κ*B activation.	Obesity-associated inflammation	(Kim et al. 2015)
	Ileum	Male Wistar rats	*In vivo*	SSa suppresses the production of TNF-*α* and IL-6 and inhibits the nucleotide-binding oligomerization domain 2 (NOD2)/NF-*κ*B signalling pathway.	Sepsis	(Zhao et al. 2015a)
	Liver	LX-2	*In vitro*	SSa down-regulates BMP-4 expression and inhibits hepatic stellate cell activation.	Liver fibrosis	(Wang et al. 2013b)
	Macrophages	RAW 264.7	*In vitro*	SSa regulates inflammatory mediators and suppresses the MAPK and NF-*κ*B signalling pathways.	Lipopolysaccharide (LPS) -induced inflammation	(Zhu et al. 2013)
	Macrophages	RAW264.7	*In vitro*	SSa inhibits receptor activator of the nuclear factor-*κ*Bligand (RANKL)-induced I*κ*B*α* phosphorylation, p65phosphorylation and NF-κB luciferase activity	Osteoporosis	(Zhou et al. 2015)
	Vascular tissue	HUVECs	*In vitro*	SSa dose-dependently inhibits the production of ROS,TNF-*α*, IL-8, COX-2 and iNOS in LPS-stimulated HUVECs.	Oxidative damage	(Fu et al. 2015)
	Liver	HSC-T6	*In vitro*	SSa decreases the expressions of ERK1/2, PDGFR, TGF-*β*1R, *α*-smooth muscle actin, and connective tissue growth factor to inhibit proliferation and activation of HSCs.	Liver inflammation and fibrogenesis	(Chen et al. 2013b)
	Macrophages	RAW264.7	*In vitro*	SSa inhibits the activation of NF-*κ*B, iNOS, COX-2 and pro-inflammatory cytokines TNF-*α* and IL-6.	LPS-induced inflammation	(Lu et al. 2012a)
	Inflammatory tissue	HMC-1	*In vitro*	SSa decreases the expression of IL-6, IL-1β and TNF-*α* and suppresses NF-*κ*B signal pathway.	Anti-inflammation	(Han et al. 2011)
	Liver	Sprague-Dawley rats	*In vivo*	SSa inhibits the expression of hepatic proinflammatory cytokines and NF-*κ*B signal pathway and increases the expression of anti-inflammatory cytokine IL-10.	Inhibition of liver injury	(Wu et al. 2008, 2010)
	Human monocytic leukemia cells	THP-1	*In vitro*	SSa inhibits oxLDL-induced activation of AKT and NF-kappaB, assembly of NLRP3 inflammasome and production of pro-inflammatory cytokines.	Atherosclerosis	(He et al. 2016)
Neuroregulation	Hippocampal tissue	Sprague-Dawley rats	*In vivo*	SSa inhibits NMDA receptor current and persistent sodium current.	Epilepsy	(Yu et al. 2012)
	CA1 neurons	Sprague-Dawley rats	*In vivo*	SSa exerts selectively enhancing effects on I A.	Epilepsy	(Xie et al. 2013)
	Spinal cord tissues	Chronic constriction injury rats	*In vivo*	SSa inhibits the activation of p38 MAPK and NF-*κ*B signalling pathways in spinal cord.	Chronic constriction injury	(Zhou et al. 2014)
	Hippocampus	Sprague-Dawley rats	*In vivo*	SSa attenuates cocaine-reinforced behaviour throughactivation of GABA(B) receptors.	Morphine-reinforced behaviour	(Yoon et al. 2012, 2013)
	Nervous tissue	Sprague-Dawley rats	*In vivo*	SSa counteracts the inflammatory response and neurological function deficits via an anti-inflammatory response and inhibition of the MAPK signalling pathway.	Nerve injury	(Mao et al. 2016)
	Nervous tissue	Sprague-Dawley rats	*In vivo*	SSa inhibits this addiction by regulating GABA(B) receptor system.	Drug addiction	(Maccioni et al. 2016)
Antitumor activity	Different cancer cells	A549, SKOV3, HeLa and Siha	*In vitro*	SSa sensitizes cancer cells to cisplatin through ROS -mediated apoptosis.	Cancer cell cytotoxicity	(Wang et al. 2010a)
	Glioma	C6 glioma cells	*In vitro*	SSa enhances the enzymatic activities of GS and CNP.	C6 glioma cells proliferation	(Tsai et al. 2002)
Antiviral activity	Human fetal lung fibroblasts	Human coronavirus 229E	*In vitro*	SSa intervenes in the early stage of viral replication, such as absorption and penetration.	Coronavirus infection	(Cheng et al. 2006)
	Lung tissue	Influenza A virus infected A549	*In vitro*	SSa attenuates viral replication, aberrant pro-inflammatory cytokine production and lung histopathology.	Pathological influenza virus infections	(Chen et al. 2015)
Immunoregulation	Lymphoid tissue	Sprague-Dawley rats	*In vivo*	SSa inhibits the proliferation and activation of T cells and causes the G0/G1 arrest as well as the induction of apoptosis via mitochondrial pathway.	Inflammatory andautoimmune diseases	(Sun et al. 2009)

### Anti-inflammatory activity

**Figure 4.**
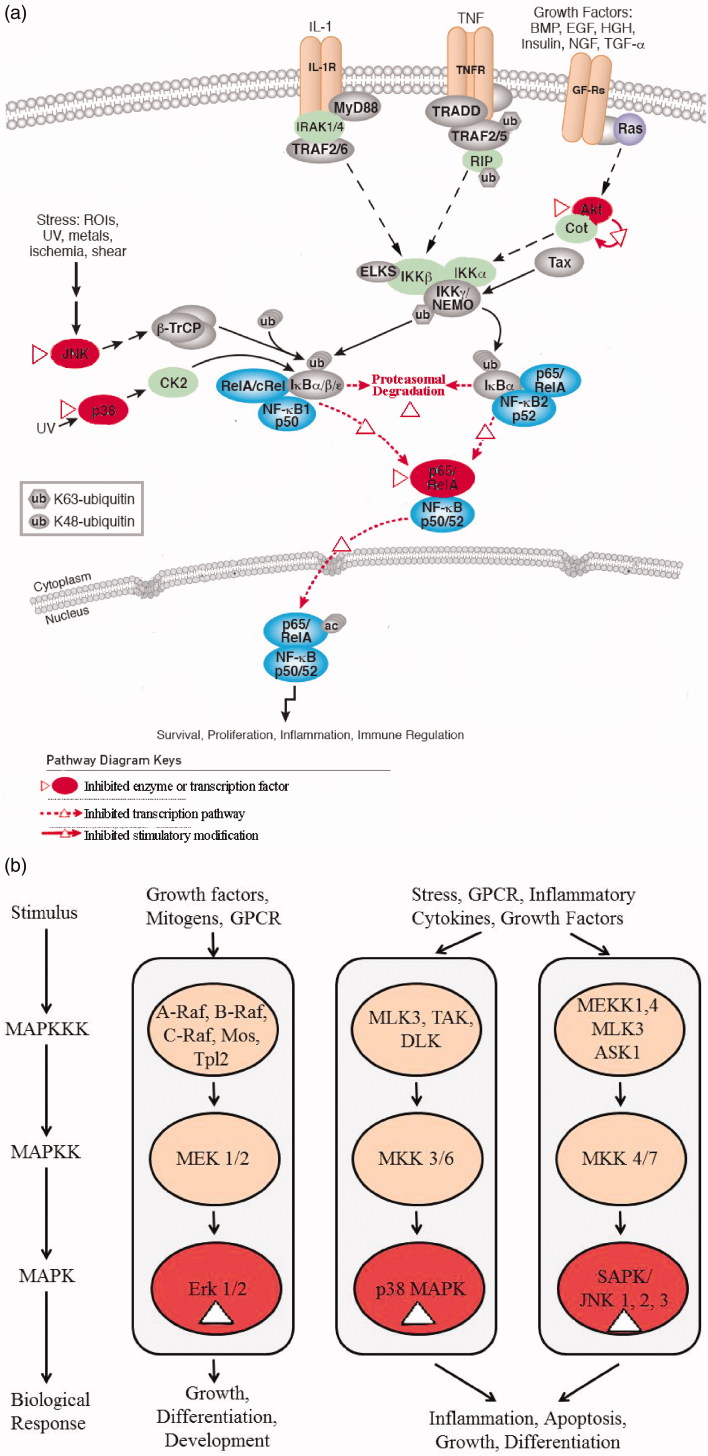
The molecular mechanisms of the anti-inflammatory activity of SSa. (a) shows the NF-*κ*B pathway, (b) shows the MAPK pathway.

Among all of the pharmacological activities of SSa, the most important one is anti-inflammatory activity. SSa develops its anti-inflammatory activity mainly by inhibiting some inflammation-associated cytokines, proteins and enzymes, and regulating inflammation-related signal pathways, such as nuclear factor-κB (NF-κB) pathway and mitogen-activated protein kinase (MAPK) pathway. In order to better explain the molecular mechanisms of the anti-inflammatory activity of SSa, Figures 4(a,b) are provided to describe its NF-κB pathway and MAPK pathway.

In general, SSa inhibits the expression of pro-inflammatory cytokines, including tumor necrosis factor α (TNF-α), transforming growth factor-β1R (TGF-β1R), interleukin 1β (IL-1β), IL-6, and IL-8, and increases the expression of anti-inflammatory cytokine TGF-β1 and IL-10 (Wu et al. 2008, 2010; Han et al. 2011; Lu et al. 2012a; Zhu et al. 2013; Fu et al. 2015; Kim et al. 2015; Zhao et al. 2015a). SSa exerts inhibiting effect on inflammatory associated proteins and enzymes, such as inducible nitric oxide synthase (iNOS), cyclooxygenase-2 (COX-2) (Lu et al. 2012b; Zhu et al. 2013; Fu et al. 2015; Kim et al. 2015), extracellular matrix-regulated kinase (ERK), c-Jun N-terminal kinase (JNK) (Chen et al. 2013b; Zhu et al. 2013; Zhou et al. 2015), and it also suppresses particular proteins, bone morphogenetic protein 4 (BMP-4) (Wang et al. 2013b), platelet-derived growth factor receptor 1 (PDGFR1) (Chen et al. 2013b) and toll-like receptor 4 (TLR4) (Fu et al. 2015) to exert particular effects (Wang et al. 2013b).

NF-*κ*B pathway is an important signal pathway in inflammatory process (Bannon et al. 2015). SSa attenuates inflammation mainly by inhibiting the activation of NF-*κ*B pathway (Wu et al. 2008, 2010; Han et al. 2011; Lu et al. 2012a; Kim et al. 2015; Zhao et al. 2015a; Zhou et al. 2015). As shown in Figure 4(a), these inhibition effects are mainly reflected in two ways. One is inhibitory effects on phosphorylation of kinases, including I*κ*B*α*, p65 (Zhu et al. 2013; Kim et al. 2015; Zhou et al. 2015), p38 (Han et al. 2011; Chen et al. 2013b; Zhou et al. 2015), JNK (Zhu et al. 2013; Zhou et al. 2015), and Akt (He et al. 2016), and the other is blocking translocation of nuclear factors, including NF-*κ*B (Lu et al. 2012a; Zhu et al. 2013; Kim et al. 2015) and NF-*κ*B/Rel A(Han et al. 2011). The above two inhibition effects are marked by triangle in Figure 4(a).

As shown in Figure 4(b), SSa also has an inhibiting effect on MAPK pathway. It downregulates the phosphorylation of three key kinase, p38 MAPK, c-JNK, and ERK 1/2, which are located in the downstream of MAPK pathway and marked by triangle symbol in Figure 4(b).

For studying the anti-inflammatory activity of SSa, it has been applied to mouse macrophage cells RAW264.7 (Zhou et al. 2015), human umbilical vein endothelial cells (HUVECs) (Fu et al. 2015), mouse embryonic fibroblasts 3T3-L1 (Kim et al. 2015), hepatic stellate cells (HSCs) (Chen et al. 2013b), and human mast cells (HMCs) (Han et al. 2011) *in vitro*, and has been applied to the livers of Sprague-Dawley rats (Wu et al. 2010) and Wistar rats (Zhao et al. 2015a) *in vivo*.

### Neuroregulation activity

SSa plays a significant role on neuroregulation. It exerts antiepileptic mainly by inhibiting *N*-methyl-D-aspartic acid (NMDA) receptor current, persistent sodium current (Yu et al. 2012) and inactivating K^+ ^current (Xie et al. 2013). It inhibits the activation of p38 MAPK, NF-*κ*B signaling pathways to attenuate neuropathic pain (Zhou et al. 2014), and activates *γ*-aminobutyric acid (GABA) receptor B to attenuate cocaine-reinforced behavior (Yoon et al. 2012, 2013) and drug addiction (Maccioni et al. 2016). It also counteracts the inflammatory response and neurological function deficits via an anti-inflammatory response and inhibition of the MAPK signaling pathway to ease nerve injury (Mao et al. 2016). SSa has been applied to the hippocamp, CA1 neurons, and spinal cord tissues of Sprague-Dawley rats (Mao et al. 2016; Maccioni et al. 2016; Yu et al. 2012; Xie et al. 2013; Yoon et al. 2012, 2013), and chronic constriction injury rats (Zhou et al. 2014) *in vivo*, which determined its potential application in epilepsy, chronic constriction injury, nerve injury, and drug addiction.

### Anti-tumor activity

SSa exhibits antitumor activity *in vitro* by sensitizing cancer cells to cisplatin, such as human lung adenocarcinoma cells A549, ovarian cancer cells SKOV3, and cervix cancer cells Hela and Siha, through reactive oxygen species (ROS)-mediated apoptosis (Wang et al. 2010a) and enhancing the enzymatic activities of glutamine synthetase (GS) and 2′,3′-cyclic nucleotide 3′-phosphohydrolase (CNP) in rat C6 glioma cells (Tsai et al. 2002). Thus, the combination of SSa with cisplatin could be an effective therapeutic strategy against cancer.

### Antiviral activity

SSa has generally inhibitory effects against human coronavirus 229E (Cheng et al. 2006) and influenza A virus (Chen et al. 2015). It exerts antiviral activity mainly through interference in the early stage of viral replication, such as absorption and penetration (Chen et al. 2015), and attenuating aberrant pro-inflammatory cytokine production (Cheng et al. 2006). These two viruses are cultured in human cells, human fetal lung fibroblasts MRC-5 and A549 cells, respectively.

### Immunoregulation activity

SSa inhibits the proliferation and activation of T cells and causes the G0/G1 cells arrest as well as the induction of apoptosis via mitochondrial pathway to exhibit its immunoregulation effect in Sprague-Dawley rats (Sun et al. 2009). This may herald a novel approach for further studies of SSa as a candidate for the treatment of autoimmune diseases.

## SSd

SSd is the epimer of SSa, they have the same basal structure. So, it has some similar pharmacological activities with SSa, such as anti-inflammatory (Lu et al. 2012b), antitumor (Chen et al. 2013a), and immunoregulation activities (Sun et al. 2009; Ying et al. 2014). However, SSd also possesses some specific pharmacological activities, such as anti-allergic (Hao et al. 2012) and anti-apoptosis activities (Li et al. 2014b). The various pharmacological activities, mechanisms, models and applications of SSd are listed in Table 2.

**Table 2.** The various pharmacological activities, mechanisms, models, and applications of SSd.

Pharmacological activities of SSd	Tissue	Models/cells	*In vivo*/*vitro*	Mechanisms	Applications	References
Antitumor activity	Liver	Sprague Dawley rats	*In vivo*	SSd inhibits the activation of CCAAT/enhancer binding protein *β* (C/EBP*β*) and COX-2.	Human hepatocellular carcinoma	(Lu et al. 2012b)
	Thyroid	ARO, 8305C, SW1736	*In vitro*	SSd promotes cell apoptosis and induced G1-phase cell cycle arrest.	Human undifferentiated thyroid carcinoma	(Liu & Li 2014)
	Liver	SMMC7721	*In vitro*	SSd suppresses the expression of COX-2 through the p-STAT3/hypoxia inducible factor-1*α* (HIF-1α) pathway.	Human hepatocellular carcinoma	(He et al. 2014)
	Prostate carcinoma cells	DU145	*In vitro*	SSd has effects on induction of apoptosis and cell cycle arrest at G0/G1 phase.	Prostate carcinoma	(Yao et al. 2014)
	Different cancer cells	HeLa, HepG2	*In vitro*	SSd suppresses TNF-*α*-induced NF-*κ*B activation and its target genes expression to inhibit cancer cell proliferation, invasion, angiogenesis and survival.	As a combined adjuvant remedy with TNF- α for cancer patients	(Wong et al. 2013a)
	Lung carcinoma	A549	*In vitro*	SSd induces apoptosis and blocked cell cycle progression by activating Fas/FasL pathway in the G1 phase in A549 cells.	Human non-small cell lung cancer	(Hsu et al. 2004a)
	Liver	HepG2, 2.2.15	*In vitro*	SSd induces the apoptosis through the activation of caspases-3 and caspases-7.	Human hepatocellular carcinoma	(Chiang et al. 2003)
	Liver	Hep3B	*In vitro*	SSd induces apoptosis in Hep3B cells through the caspase-3 -independent pathways.	Human hepatocellular carcinoma	Zhou 2003
	Breast carcinomas tissue	MCF-7	*In vitro*	SSd activates oestrogen response element (ERE)-luciferase activity via the ER *α*-mediated pathway.	Acting as a weak phytoestrogen.	(Wang et al. 2010a)
	Liver	SMMC-7721, HepG2	*In vitro*	SSd has a radiosensitizing effect on hepatoma cells under hypoxic conditions by inhibiting HIF-1*α* expression.	Radiotherapy sensitizer in hepatoma radiotherapy	(Wang et al. 2014a, 2014b)
	Different cancer cells	HeLa, MCF-7	*In vitro*	SSd induces autophagy through the formation of autophagosomes by inhibiting SERCA.	Apoptosis-resistant cancer cells	(Wong et al. 2013b)
Anti-inflammatory activity	Inflammatory tissue	RAW264.7	*In vitro*	SSd has inhibitory effects on NF-*κ*B activation and iNOS, COX-2 and pro-inflammatory cytokines including TNF-*α* and IL-6.	LPS-induced inflammation	(Lu et al. 2012a)
	Hepatic stellate cells	HSC-T6	*In vitro*	SSd decreases the expressions of extracellular matrix-regulated kinase 1/2 (ERK1/2), PDGFR, TGF-*β*1R, *α*-smooth muscle actin, TGF-*β*1 and connective tissue growth factor.	Liver inflammation and fibrogenesis	(Chen et al. 2013a)
	Human acute monocytic leukaemia cells	THP-1	*In vitro*	SSd inhibits selectin-mediated cell adhesion.	L-selectin-mediated cell adhesion	(Jang et al. 2014)
	Liver	C57/BL6 rats	*In vivo*	SSd down-regulates NF-*κ*B and STAT3-mediated inflammatory signal pathway.	Hepatotoxicity and liver injury	(Liu et al. 2014a)
	Liver	Hepatic fibrosis rats	*In vivo*	SSd down-regulates liver TNF-*α*, IL-6 and NF-*κ*B p65 expression and increases I*κ*B-*α* activity.	Hepatic fibrosis	(Dang et al. 2007)
	Kidney	LLC-PK1	*In vitro*	SSd increases the activity and expression of anti-oxidant enzymes (SOD, CAT, GPx) and HSP72.	Oxidative damage in the kidney	(Zhang et al. 2014)
	Nervous tissue	C6 rat glioma cells	*In vitro*	SSd possesses a dual effect: an inhibition of PGE2 production without a direct inhibition of cyclooxygenase activity and an elevation of [Ca^2+^]i.	Inflammation in C6 rat glioma cells	(Kodama et al. 2003)
	Lung	VILI rats	*In vivo*	SSd decreases the expression of pro-inflammatory cytokines including MIP-2, IL-6 and TNF-*α* and elevates the expression of anti-inflammatory mediators, such as TGF-*β*1 and IL-10.	Lung injury	(Wang et al. 2015)
	Renal tubular epithelial cells	NRK-52E	*In vitro*	SSd attenuates oxidative injury via upregulation of SirT_3_.	High glucose induced kidney injury	(Zhao et al. 2015b)
	Kidney	HK-2	*In vitro*	SSd represses ROS-mediated activation of MAPK and NF-*κ*B signal pathways.	DDP-induced kidney injury	(Ma et al. 2015)
Immunoregulation	Lymphoid tissue	Mouse T cells	*In vitro*	SSd inhibits the T cell proliferation and activation through the NF-*κ*B, NF-AT and AP-1 signal pathways, and it also inhibits the cytokine secretion and IL-2 receptor expression.	T cell-mediated autoimmune conditions	(Wong et al. 2009)
	Monocyte-derived dendritic cells	DCs	*In vitro*	SSd reduces the differentiation of human DCs and promotes DCs maturation and increases the function of mature DCs.	Condylomata acuminata	(Ying et al. 2014)
Anti-allergic activity	Lymphoid tissue	Rat basophilic leukemia-2H3 cells	*In vitro*	SSd suppresses the intracellular calcium mobilization and tyrosine phosphorylation, thereby prevents gene activation of Cdc42 and c-Fos.	Soybean allergy	(Hao et al. 2012)
Neuroregulation	Neuronal cells	PC12	*In vitro*	SSd regulates mitochondrial and nuclear GR translocation, partial reversal of mitochondrial dysfunction, inhibition of the mitochondrial apoptotic pathway, and selective activation of the GR-dependent survival pathway.	Against corticosterone-induced apoptosis	(Li et al. 2014b)
	Neuronal cells	PC12	*In vitro*	SSD reduces PC12 cells apoptosis by removing ROS and blocking MAPK-dependent oxidative damage.	Neuronal oxidative stress	(Lin et al. 2016)

### Antitumor activity

**Figure 5.**
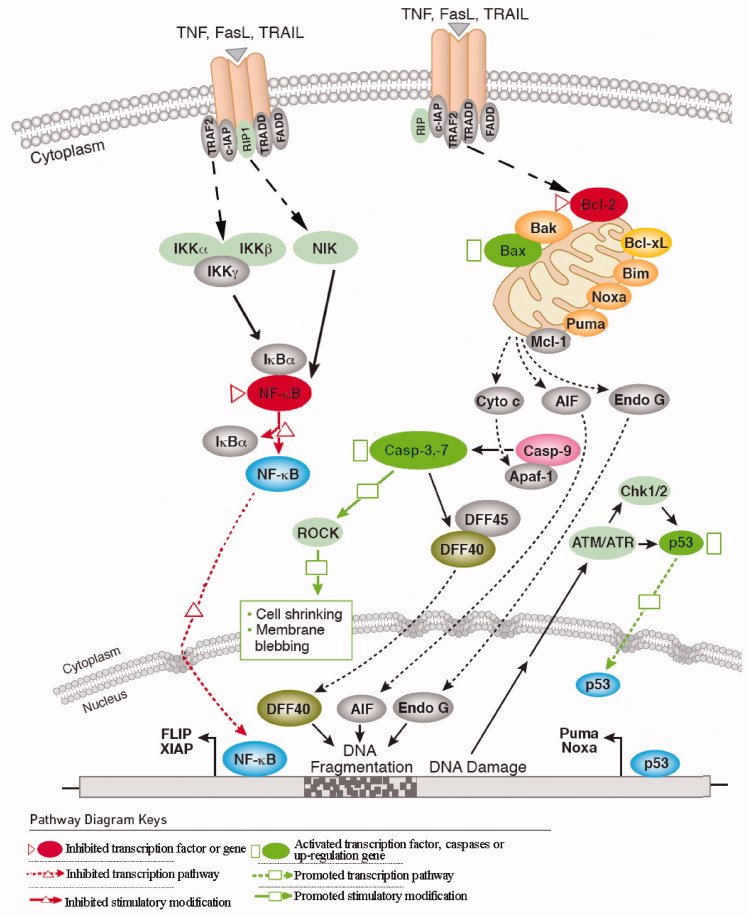
The molecular mechanisms of the anti-tumor activity of SSd.

The most important pharmacological activity of SSd is antitumor activity. In order to better explain this important activity, Figure 5 is provided to describe its molecular mechanisms. SSd exhibits the antitumor activity mainly through activation and inhibition, which are marked by rectangle and triangle in Figure 5, respectively. First, SSd increases the expression of p53 and Bax (Liu & Li 2014; Wang et al. 2014a, 2014b; Yao et al. 2014), activates caspases apoptosis pathway, including the activation of caspases-3 and caspases-7 (Chiang et al. 2003; Chou et al. 2003) and the Fas/FasL apoptotic system (Hsu et al. 2004a) in several cancer cell lines *in vitro*, which are marked by rectangle in Figure 5. Second, SSd decreases the expression of B cell lymphoma 2 (Bcl-2) family proteins (Liu & Li 2014; Wang et al. 2014a, 2014b; Yao et al. 2014), suppresses the expression of COX-2, which has been shown to be involved in carcinogenesis (Lu et al. 2012b; He et al. 2014), and also potentiates TNF-*α*-mediated cell death via suppression of TNF-*α*-induced NF-*κ*B activation (Wong et al. 2013a), which are marked by triangle in Figure 5. Besides, SSd also suppresses MCF-7 cells proliferation through the estrogenic effect of SSd by the estrogen receptor (Wang et al. 2010a, 2010b), and induces autophagy of apoptosis-resistant cancer cells through the formation of autophagosomes by inhibiting sarcoplasmic/endoplasmic reticulum Ca^2+ ^ATPase pump (SERCA) (Wong et al. 2013b).

To date, SSd has been applied in human hepatoma cells HepG2, Hep3B (Chou et al. 2003), SMMC7721 (He et al. 2014), and 2.2.15 cells (Chiang et al. 2003), anaplastic thyroid cancers cells ARO, 8305C, and SW1736 (Liu & Li 2014), prostate carcinoma cells DU145 (Yao et al. 2014), lung cancer cells A549 (Hsu et al. 2004a), cervical carcinoma cells Hela (Wong et al. 2013a, 2013b), and breast carcinoma cells MCF-7 (Wang et al. 2010b) *in vitro*, and applied in diethylinitrosamine (DEN)-treated Sprague Dawley rats *in vivo* (Lu et al. 2012b), and which indicates its potential in treatment of cancer.

### Anti-inflammatory activity

SSd also possesses an evident anti-inflammatory activity, and the mechanisms are similar to SSa, as shown in Figure 4(a). On the cytokines level, SSd suppresses pro-inflammatory cytokines including TNF-α, IL-6, macrophage inflammatory protein-2 (MIP-2), and elevates the expression of anti-inflammatory cytokines, such as TGF-β1 and IL-10 (Lu et al. 2012a; Ma et al. 2015; Wang et al. 2015). On the level of proteins and enzymes, it inhibits the activity and expression of iNOS, COX-2, ERK1/2, PDGFR, α-smooth muscle actin, NF-*κ*B, and signal transducer and activator of transcription 3 (STAT3) (Chen et al. 2013a; Liu et al. 2014a), and increases the activity and expression of inhibitor of nuclear factor of *κ*B-*α* (I*κ*B-*α*) (Dang et al. 2007), SirT3 (Zhao L et al. 2015), anti-oxidant enzymes (superoxide dismutase (SOD), catalase (CAT), glutathione peroxidase (GPx) and heat shock protein (HSP) 72 (Zhang et al. 2014). Furthermore, SSd also exhibits its particular anti-inflammatory pattern by inhibiting selectin-mediated cell adhesion (Jang et al. 2014), and possessing a dual effect, an inhibition of prostaglandin E_2_ (PGE_2_) production without a direct inhibition of cyclooxygenase activity and an elevation of Ca^2+^ (Kodama et al. 2003).

According to the above reports, SSa and SSd are very similar in mechanisms of anti-inflammation, however, there are still several different points, which are listed in Table 3. SSa is able to inhibit phosphorylation of three key kinase in MAPK pathway, which was not reported in researches of SSd. While SSd is able to restrain selectin-mediated cell adhesion, PGE_2_ production, and elevate the Ca^2+ ^level intracellular, which were not reported in researches of SSa

**Table 3.** The similarities and differences of SSa and SSd in mechanisms of anti-inflammation.

The possible mechanisms of anti-inflammation	SSa	SSd
Inhibiting pro-inflammatory cytokines and promoting anti-inflammatory cytokines	✓	✓
Inhibiting activity of enzymes associated with inflammation	✓	✓
Inhibiting activation of NF-κB pathway	✓	✓
Inhibiting activation of MAPK pathway	✓	✗
Inhibiting selectin-mediated cell adhesion	✗	✓
Inhibiting PGE2 production and elevating Ca^2+^ level intracellular	✗	✓

For a better understanding of SSd’s anti-inflammatory activity, it has been applied to mouse leukaemic monocyte macrophage macroph RAW264.7 (Lu et al. 2012a), hepatic stellate cells HSC-T6 (Chen et al. 2013a), human acute monocytic leukemia cells THP-1 (Jang et al. 2014), pig kidney proximal tubular cells LLC-PK1 (Zhang et al. 2014), C6 rat glioma cells (Kodama et al. 2003), renal tubular epithelial cells NRK-52E (Zhao et al. 2015b), and HK-2 (Ma et al. 2015) *in vitro*, and acetaminophen-induced hepatotoxicity C57/BL6 rats (Liu et al. 2014a), hepatic fibrosis model rats (Dang et al. 2007), and ventilator-induced lung injury (VILI) rats (Wang et al. 2015) *in vivo*, which determined its potential application for treating hepatitis, pneumonia, nephritis and other inflammation.

### Immunoregulation activity

SSd plays its immunoregulation role by regulating the NF-*κ*B, nuclear factor-AT (NF-AT), and activator protein 1 (AP-1) signal pathways to inhibit T cell proliferation and activation (Wong et al. 2009). It has been applied to condylomata acuminate, a disease caused by human papilloma virus (HPV), by reducing the differentiation of human monocyte-derived dendritic cells (DCs) and promoting DCs maturation and increasing the function of mature DCs (Ying et al. 2014).

### Anti-allergic activity

β-Conglycinin has been identified as a potential diagnostic marker for severe basophil-dependent allergic reactions to soybean. SSd possesses anti-allergic activity by inhibiting β-conglycinin-induced rat basophilic leukemia-2H3 cell degranulation and suppressing critical incidents in the signal transduction pathway (Hao et al. 2012), Hence it could become an effective herbal therapy for alleviating soybean allergy.

### Neuroregulation activity

Neuronal oxidative stress injury has been proven to be associated with many neurodegenerative diseases. SSd exerts neuroregulation activity on neuronal PC12 cells by inhibiting the translocation of the glucocorticoid receptor (GR) to the mitochondria, restoring mitochondrial function, down-regulating the expression of pro-apoptotic-related signalling events and up-regulating anti-apoptotic-related signalling events (Li et al. 2014b). In H_2_O_2_-induced oxidative stress PC12 cells, SSd effectively decreases oxidative stress injury by blocking H_2_O_2_-induced phosphorylation of ERK, JNK, and p38MAPK to exert neuroregulation activity (Lin et al. 2016). Thus, SSd treatment is an effective method for treating neurodegenerative diseases.

## SSc

SSc has the same basal structure with SSa and SSd. They are epoxy-ether saikosaponins belonging to type I saikosaponins (Shin et al. 2015). However, the pharmacological activities of SSc are far weaker than SSa and SSd. To date, reports about pharmacological activities of SSc are very limited. SSc exerts anti-apoptotic effects on HUVECs by suppressing caspase-3 activation and subsequent degradation of focal adhesion kinase (FAK) and other cell adhesion signals, which is similar to SSa (Lee et al. 2014). Thus, it will be a promising therapeutic candidate for the treatment of vascular endothelial cell injury and cellular dysfunction. Besides, SSc completely prevents the development of nephritis (Chen et al. 2008), but the mechanism of this activity is still unclear. In addition, SSc exhibits antiviral activity by inhibiting hepatitis B virus (HBV) DNA replication (Chiang et al. 2003).

## SSb_2_

SSb_2_ has a different basic structure compared to SSa, SSd, and SSc. SSb_2_ is a type II saikosaponin, and it is not considered as a main active compound in Radix Bupleuri. However, SSb_2_ has fairly inhibitory effects against corona virus and hepatitis C virus (HCV). It mainly interferes with the early stages of viral replication, such as absorption and penetration of the virus (Cheng et al. 2006). SSb_2_ potently inhibits HCV infection at non-cytotoxic concentrations through efficient inhibition on early HCV entry, including neutralization of virus particles, preventing viral attachment, and inhibiting viral entry/fusion (Lin et al. 2014).

## Radix Bupleuri extracts

Many *Bupleurum* medicinal plants are used as Radix Bupleuri. The pharmacological activities of extracts from seven *Bupleurum* species, *B. chinense* (Wen et al. 2011), *B. falcatum* (Lee et al. 2012a), *Bupleurum marginatum* Wall. ex DC. (Ashour et al. 2014), *B. yinchowense* (Li et al. 2013), *Bupleurum kaoi* L. (Hsu et al. 2004a, 2004b), *B. scorzonerifolium* (Cheng et al. 2005), and *Bupleurum longiradiatum* Turcz. (You et al. 2002), are given in Table 4. They have been demonstrated to possess antitumor (Cheng et al. 2003, 2005; Hsu et al. 2004a, 2004b; Chen et al. 2005; Kang et al. 2008; Ashour et al. 2014), antiviral (Wen et al. 2011), anti-inflammatory (Lee et al. 2010; Nakahara et al. 2011), anti-hyperthyroidism (Kim et al. 2012b) and neuroregulation effects (Xie et al. 2006; Lee et al. 2009, 2012b; Li et al. 2013; Liu et al. 2014b).

**Table 4.** The pharmacological activities and mechanisms of extracts from different *Bupleurum* species.

Species	Extractive fractions	Extraction method	Activities	Mechanisms	References
*B. chinenes*	Aqueous extracts	Water decoction, 3 h	Antitumor activity	Enhancing 5-fluorouracil-induced cytotoxicity in HepG2 hepatoma cells and protecting normal blood lymphocytes.	(Kang et al. 2008)
		Water decoction, 3 h	Antiviral activity	Suppressing the effect on regulated activation normal T-cell expressed (RANTES) secretion.	(Wen et al. 2011)
		Water decoction, 3 h	Affect drug distribution	Inhibiting the activity of β-glucuronidase.	(Chen et al. 2014)
	Methanol TSS extracts	Methanol, reflux, 4 h	Neuroregulation	Suppressing the abnormal activation of hippocampal astrocyte through inhibiting the overexpression of glial fibrillary acidic protein.	(Xie et al. 2006)
		95% methanol 5% pyridine, reflux, 4 h		TSS antagonizes the reserpine-induced akinesia, and ptosis in mice.	(Liu et al. 2014a)
*B. falcatum*	Ethanol extracts	70% ethanol, reflux, 6 h	Anti-inflammatory activity	Inhibiting the expression and activation of both metal matrix proteinase (MMP)-2 and MMP-9 after spinal cord injury (SCI) and the mRNA expressions of TNF-*α*, IL-1*β*, COX-2, and iNOS.	(Lee et al. 2010)
		80% ethanol, reflux, 6 h	Anti-depressant activity	Reducing depression and anxiety-like behaviors, possibly through central adrenergic mechanism.	(Lee et al. 2012a)
		80% ethanol, reflux, 6h	Memory improvement	Attenuating IMO stress-induced loss of cholinergic immunoreactivity in the hippocampus.	(Lee et al. 2009)
	Methanol extracts	Methanol, reflux, 4 h	Anti-depressant activity	The mechanism of this activity involves the serotonergic and noradrenergic systems.	(Kwon et al. 2010)
		Methanol, reflux, 4 h	Anti-inflammatory activity	Decreasing the content of alanine transaminase (ALT) in blood serum of the liver injury rats.	(Nakahara et al. 2011)
	Aqueous extracts	Water decoction, 3 h	Anti-hyperthyroidism	Attenuating LT4-induced hyperthyroidisms and normalizing LT4-induced liver oxidative stresses and reducing liver and epididymal fat pad changes.	(Kim et al. 2012b)
*B. scorzonerifolium*	Acetone extracts	Acetone, reflux, 4 h	Antitumor activity	Inducing tubulin polymerization, and activates caspase-3 and caspase-9 in A549 cells, and these effects are related to ERK 1/2 activation and the apoptosis.	(Chen et al. 2005; Cheng et al. 2005)
		Acetone, reflux, 4 h		Inhibiting telomerase activity and activation of apoptosis.	(Cheng et al. 2003)
*B. marginatum*	Methanol extracts	Methanol, reflux, 6 h	Anti-infective and antitumor activities	Methanol extracts show a significant anti-trypanosomal activity and moderate activity against *Streptococcus pyogenes* and have the cytotoxicity inducing apoptosis.	(Ashour et al. 2014)
*B. longiradiatum*	Ethyl acetate extracts	Ethyl acetate, reflux, 4 h	Antiangiogenic activity	It has an inhibitory effect on the tube-like formation of HUVECs.	(You et al. 2002)
*B. yinchowense*	Ethanol TSS extracts	60% ethanol 0.5% ammonia reflux, 6 h	Neuroregulation	The neuroprotective mechanism relates with inhibiting the ER stress and the mitochondrial apoptotic pathways.	(Li et al. 2013)
*B. kaoi*	Methanol TSS extracts	Methanol, reflux, 4 h	Antitumor activity	The activity of the Fas/Fas ligand apoptotic system participates in the antiproliferative activity of TSS in A549 cells.	(Hsu et al. 2004b)
		Methanol, reflux, 4 h		Extracts from *B. kaoi* show potent antiproliferative effects on human A375.S2 melanoma cells.	(Hu et al. 2016)

Five kinds of extraction agents, water, methanol, ethanol, acetone and ethyl acetate, have been used to extract effective fractions from Radix Bupleuri. Aqueous extracts of Radix Bupleuri are obtained by boiling at 80 °C for 3 h, and then evaporating and lyophilizing (Kang et al. 2008; Wen et al. 2011; Kim et al. 2012b; Chen et al. 2014). The method to obtain methanol, ethanol, acetone and ethyl acetate extracts is reflux extraction (You et al. 2002; Cheng et al. 2005; Lee et al. 2010; Liu et al. 2014a). To obtain methanol extracts, Radix Bupleuri is extracted twice by 100% methanol or 95% methanol with 5% pyridine at 70 °C for 4 h (Xie et al. 2006; Kwon et al. 2010; Nakahara et al. 2011; Liu et al. 2014a; Ashour et al. 2014). To obtain ethanol extracts, Radix Bupleuri is extracted twice by 60% (Li et al. 2013), 70% (Lee et al. 2010) or 80% ethanol (Lee et al. 2012a) at room temperature for 6 h. To obtain acetone and ethyl acetate extracts, Radix Bupleuri is extracted three times by 100% acetone and 100% ethyl acetate at room temperature for 4 h (You et al. 2002; Cheng et al. 2005).

The pharmacological activities of extracts from *B. chinense* and *B. falcatum* have relative in-depth studies. The aqueous extracts of *B. chinense* possess three activities, antitumor activity on HepG2 hepatoma cells (Kang et al. 2008), antiviral activity on H1N1-infected A549 cells (Wen et al. 2011), and an activity to affect drug distribution (Chen et al. 2014). Methanol total saikosaponins (TSS) extracts of *B. chinense* have a neuroregulation effect (Xie et al. 2006; Liu et al. 2014a). In chronic kindling rats induced by pentetrazole (PTZ), TSS of *B. chinense* inhibit glial fibrillary acidic protein (GFAP) over-expression and suppress the abnormal activation of hippocampal astrocyte (Xie et al. 2006). Anti-depressant activity of TSS is investigated by tail suspension test, forced swimming test, and reserpine antagonism test in mice, which demonstrate that it shortens the immobility time of mice in the tail suspension test in a somewhat dose-dependent manner (Liu et al. 2014a).

Both ethanol extracts and methanol extracts of *B. falcatum* have an anti-inflammatory effect (Lee et al. 2010; Nakahara et al. 2011) with similar mechanisms to SSa. They also possess an anti-depressant activity possibly through central adrenergic mechanism (Kwon et al. 2010; Lee et al. 2012a). Besides, the ethanol extracts of *B. falcatum* has its specific memory improvement activity by attenuating immobilization (IMO) stress-induced loss of cholinergic immunoreactivity in the hippocampus (Lee et al. 2009). The aqueous extracts of *B. falcatum* has an anti-hyperthyroidism activity by attenuating leukotriene-4 (LT4)-induced hyperthyroidisms, normalizing LT4-induced liver oxidative stresses and reducing liver and epididymal fat pad changes (Kim et al. 2012b).

The acetone extracts of *B. scorzonerifolium* exerts stronger antitumor activity on A549 cells mainly through inducing tubulin polymerization (Chen et al. 2005), activating caspase-3 and caspase-9 (Cheng et al. 2005), and inhibiting telomerase activity and activation of apoptosis (Cheng et al. 2003). Methanol extracts of *B. marginatum* and *B. kaoi* have an antitumor activity by inducing apoptosis (Ashour et al. 2014) and activating the Fas/Fas ligand apoptotic system respectively (Hsu et al. 2004b), and extracts of *B. kaoi* have antitumor activity on human A375.S2 melanoma cells by inhibiting phosphorylation of JNK, p38 and p53, decreasing level of cytochrome c (Hu et al. 2016). What’s more, the ethanol TSS extracts of *B. yinchowense* show antidepressant activity by inhibiting the estrogen receptor (ER) stress and the mitochondrial apoptotic pathways (Li et al. 2013), and the ethyl acetate extracts of *B. longiradiatum* exhibit an antiangiogenic activity by inhibiting the tube-like formation of HUVECs (You et al. 2002).

## Applications of Radix Bupleuri in TCM

Radix Bupleuri has been used for more than 2000 years in China since its first record in *Shen Nong Ben Cao Jing* (Xie et al. 2009). And now, it is officially listed in *Chinese Pharmacopeia*. In TCM, Radix Bupleuri is mainly used to treat liver diseases, alleviate cold fever, chills, chest pain, regulate menstruation, and improve uterine prolapsed (Zhou 2003). In particular, Radix Bupleuri also plays a significant role in the treatment of malaria (Xue et al. 1996). Importantly, Radix Bupleuri is usually used as monarch drug in many traditional Chinese prescriptions.

To date, Radix Bupleuri has been used in about 150 traditional Chinese prescriptions. Among them, *Xiao Chai Hu Tang*, *Chai Hu Gui Zhi Tang*, and *Xiao Yao San* are very famous in TCM. *Xiao Chai Hu* decoction, including Radix Bupleuri, pinellia (the tuber of *Pinellia ternata* (Thunb.) Breit., *Banxia* in Chinese) and skullcap (the root of *Scutellaria baicalensis* Georgi, *Huangqin* in Chinese), is used to treat malaria and jaundice. When Radix Bupleuri combines with cassia twig (the twig of *Cinnamomum cassia* Presl, *Guizhi* in Chinese), it is called *Chai Hu Gui Zhi* decoction which is often used for regulating liver-*qi*, clearing heat, and lifting yang *qi*. *Xiao Yao San*, composed of Radix Bupleuri, Poria (*Poria cocos* (Schw.) Wolf), Radix Paeoniae Alba (*Paeonia lactiflora* Pall.), Radix Angelicae Sinensis (*Angelica sinensis* (Oliv.) Diels), Rhizoma Atractylodis Macrocephalae (*Atractylodes macrocephala* Koidz.), Herba Menthae (*Mentha haplocalyx* Briq.), and Rhizoma Zingiberis Recens (*Zingiber officinale* Rosc.), has been widely used in clinic for treating mental disorders, such as depression and irregular menstruation. In addition, combination with ginseng (*Panax ginseng* C.A.Mey.) and Radix Astragali (*Astragalus membranaceus* (Fisch.) Bge.). Radix Bupleuri is also used to treat hemorrhoids, anal and uterine complications, and diarrhea (1998; 1999; World Health Organization 1997). Inspired by the role in regulating metabolism and controlling *Yin*/*Yang* as mentioned in the traditional Chinese medicine, Radix Bupleuri is also widely used in Korea and Japan (Van & Wink 2004; Pan 2006).

## Applications of Radix Bupleuri in modern Chinese medicine

With the development of TCM modernization, more Radix Bupleuri preparations have been developed, such as *Xiao Chai Hu* tablets, *Chai Hu* dripping pills, *Chai Hu* injection and *Chai Hu Shu Gan* pills (Li et al. 2014a). The preparations from Radix Bupleuri approved by CFDA from June 2010 to October 2015 are given in Table 5. Among them, *Chai Hu* injection is the first successful traditional Chinese medicine injection having been used in clinic since 1940s, which is widely used to treat fever caused by influenza or common cold and malaria (Zuo et al. 2013). Moreover, some new dosage forms of Radix Bupleuri have been prepared. A nasal temperature-sensitive *in situ* gel system is developed, which is more effective for the treatment of fever than the traditional nasal spray (Chen et al. 2010). Another benefit of this novel *in situ* gel is that it exhibits more noticeable antipyretic effects and remains much more time (Cao et al. 2007). Besides, the Radix Bupleuri suppositoria is very suitable for kids without pain (Wang & Chen 2003).

**Table 5.** The preparations from Bupleuri Radix approved by CFDA.

Components	Dosage forms	China Approved Drug Names (CADN)	Batch number	Approval date	Drug standard code
Radix Bupleuri extract, poly yamanashi ester-80, sodium chloride	Injection	*Chai Hu* Injection	Z61021126	07/2013	86902434000703
Radix Bupleuri dry extract	Tablet	*Chai Hu* Cough Tablets	Z42020845	06/2015	86901876000227
Radix Bupleuri, scutellaria, pinellia, dangshen, ginger, licorice and jujube	Tablet	*Xiao Chai Hu* Tablets	Z20023393	10/2015	86903050000405
Radix bupleuri, polyethylene glycol	Dripping Pill	*Chai Hu* Dripping Pills	Z20020053	07/2015	86900941000063
Radix Bupleuri, scutellaria, pinellia, dangshen, ginger, licorice and jujube	Decoction Pill	*Xiao Chai Hu* Decoction Pills	Z41021830	06/2015	86903082001340
Radix Bupleuri, scutellaria, pinellia, dangshen, ginger, licorice, jujube	Particle	*Xiao Chai Hu* Particles	Z34020723	05/2015	86904366000721
Radix Bupleuri, scutellaria, pinellia, dangshen, ginger, licorice, jujube	Capsule	*Xiao Chai Hu* Capsules	Z20090882	08/2014	86904641002884
Radix Bupleuri, scutellaria, rhubarb, immature bitter orange, pinellia, paeoniae, jujube, ginger	Particle	*Da Chai Hu* Particles	Z20080007	02/2013	86901622002642
Radix Bupleuri, tangerine peel, ligustici, rhizoma cyperi, hoveniadulcis, paeoniae, licorice	Pill	*Chai Hu Shu Gan* Pills	Z20073333	07/2015	86901174000103
Radix Bupleuri extract	Oral Liquid	*Chai Hu* Oral Liquid	Z20020107	06/2010	86903099000244
Radix Bupleuri, sileris, tangerine peel, paeoniae, licorice, ginger	Particle	*Zheng Chai Hu Yin* Particles	Z20003013	06/2015	86901622002086
Radix Bupleuri, sileris, tangerine peel, paeoniae, licorice, ginger	Capsule	*Zheng Chai Hu Yin* Capsules	Z20040013	07/2015	86904398000362
Radix Bupleuri, sileris, tangerine peel, paeoniae, licorice, ginger	Mist	*Zheng Chai Hu Yin* Mixture	Z20090749	06/2014	86901622002666
Radix Bupleuri, scutellaria, pinellia, dangshen, ginger, licorice and jujube	Effervescent tablet	*Xiao Chai Hu* Effervescent Tablets	Z20060458	11/2011	86900042000085
Radix Bupleuri extract, acetaminophen	Injection	Paracetamol and Bupleurum Injection	H52020518	09/2010	86905510000024

## Side effects of Radix Bupleuri

Radix Bupleuri is not defined as a toxic medicine in many official pharmacopeias, such as *Chinese Pharmacopeia* and *Japanese Pharmacopeia* (National Pharmacopoeia Committee 2010; Japanese Pharmacopoeia Editorial Board 2011). However, in practical use, it exhibits liver, kidney, and blood system toxicity by taking a large dose for a long period, while it shows no side effect without over-dose (Liu et al. 2012). *Chai Hu* injection may cause a hypersensitivity-like response, hypokalemia and renal failure. And one case is reported to die from severe hypersensitivity shock (Wu et al. 2014). So, the safety of Radix Bupleuri preparations is of great concern to us.

Saikosaponins and essential oils are believed to be the main compounds responsible for side effects of Radix Bupleuri (Liu et al. 2012). Essential oils from *B. chinense* cause hepatic injury when the dosage is about 1.5–3.4 times of the clinical daily dosage of Radix Bupleuri oral liquid (Sun & Yang 2011). Saikosaponins from *B. chinense* induce the hepatoxicity by causing liver cell damage and necrosis administrating continuously to rats for 15 days (Huang et al. 2010). SSd stimulates mitochondrial apoptosis in hepatocytes to exhibit its hepatotoxicity (Chen et al. 2013a).

Extracts of Radix Bupleuri also show some side effects. Extracts of *B. chinense* induce hepatotoxicity damage through oxidative damage mechanism, and the hepatotoxicity damage caused by the alcohol extracts is more serious than that caused by aqueous extracts (Lv et al. 2009). Furthermore, LD_50_ (50% lethal dose) of the aqueous extracts of Radix Bupleuri after single oral treatment in female and male mice are considered to be over 2000 mg/kg (Kim et al. 2012a). In Kampo (Japanese traditional herbal) medicines, studies of some potential interactions between Radix Bupleuri and other drugs are considered, especially in prescriptions containing Radix Bupleuri, such as *Shosaikoto*, *Daisaikoto*, *Saikokeishito*, *Hochuekkito*, *Saibokuto* and *Saireito*. They may lead to anorexia, slight fever, and nausea (Ikegami et al. 2006).

Among other *Bupleurum* species, *B. longiradiatum* is a toxic herb in *Chinese Pharmacopeia* (National Pharmacopoeia Committee 2010), and it cannot be used as Radix Bupleuri. The main toxic compounds in *B. longiradiatum* are acetyl-bupleurotoxin, bupleurotoxin (Zhao et al. 1987) and polyene acetylene compounds, which are able to cause neurotoxicity (Chen et al. 1981).

## Discussion and perspective

Saikosaponins, especially SSa and SSd, are the main active compounds in Radix Bupleuri. They are also prescribed as the marker compounds to evaluate the quality of Radix Bupleuri in *Chinese Pharmacopeia* (National Pharmacopoeia Committee 2010). They possess evident anti-inflammatory, antitumor, neuroregulation, hepatoprotection, immunoregulation, antiviral, and antioxidative activities. And what need to emphasize is that SSa has a strongest anti-inflammatory effect, and SSd possesses a strongest antitumor effect compared with other saikosaponins, and both SSb_2_ and SSc have a better antiviral activity than SSa and SSd, which proves that the activities of different saikosaponins have some extent tendency. Inspired by this feature, we speculate that purified saikosaponin has more concentrated pharmacological activities than extracts.

Recently, more preparations containing Radix Bupleuri have been developed, such as *Xiao Chai Hu* tablets, *Chai Hu* dripping pills, *Chai Hu* injection, and *Chai Hu Shu Gan* pills (Li et al. 2014a). In these preparations the extracts of Radix Bupleuri, especially saikosaponins (Hu et al. 2011), are the main composition. Although *B. chinense* and *B. scorzonerifolium* are the only two original plants of Radix Bupleuri in *Chinese Pharmacopeia*, many other *Bupleurum* species are often used as Radix Bupleuri in China. However, the extracts of *B. chinenes*, *B. falcatum*, *B. marginatum*, *B. yinchowense*, *B. kaoi*, *B. scorzonerifolium*, and *B. longiradiatum* possess different pharmacological activities, such as the antitumor and antiviral activities of *B. chinenes* extracts, and the anti-inflammatory, anti-hyperthyroidism and neuroregulation activities of *B. falcatum* extracts. Because the quality, botanic characteristic and property, and pharmacological activities of different *Bupleurum* species are different, the standardization of Bupleuri Radix extracts is vital for the safe use of Radix Bupleuri.

In addition, there are many other compounds in Radix Bupleuri, such as polysaccharides and essential oils. Polysaccharides in Radix Bupleuri usually exert hepatoprotective and immunoregulation activities. The hepatoprotective effect of Radix Bupleuri polysaccharides is evaluated by measuring aspartate transaminase (AST), alanine transaminase, alkaline phosphatase (ALP) and lactate dehydrogenase (LDH) activities in the plasma of mice (Zhao et al. 2012), and Radix Bupleuri polysaccharides inhibits complement activation on both the classical and alternative pathways (DI HY et al. 2013). The essential oils of Radix Bupleuri have strong antimicrobial (Ashour et al. 2009) and antifungal activities (Mohammadi et al. 2014). Besides, Radix Bupleuri also contains a little lignans, which exhibit antitumor (Ou et al. 2012) and hepatoprotective activities (Lee et al. 2011, 2012). Since polysaccharides (Tong et al. 2013; Wu et al. 2013) and essential oils (Liu et al. 2009; Yan et al. 2014) have been found to possess excellent pharmacological activities so far, we suppose that the quality evaluation method should be updated to meet the need of clinical therapy.

Radix Bupleuri also exhibits some security problems in the clinic. Since ‘*Xiao Chai Hu* Decoction event’ occurred in late 1980s in Japan, the clinical safety of Radix Bupleuri has been considered (Wu et al. 2014). The reasons of toxicity are complex and there is a great individual variation in the susceptibility to Radix Bupleuri. The current researches have shown that the toxicity of Radix Bupleuri mainly associated with dosage and drug administration time (Liu et al. 2012). For example, SSd exhibits antitumor activity on carcinoma cell lines with dose-dependence, but when the dosage of SSd increased to a high level it would exert cytotoxicity (Zhang et al. 2015). Usually, Radix Bupleuri is believed to be safe in defined dose prescribed by pharmacopeia.
